# Negative Differential Resistance of n-ZnO Nanorods/p-degenerated Diamond Heterojunction at High Temperatures

**DOI:** 10.3389/fchem.2020.00531

**Published:** 2020-07-15

**Authors:** Dandan Sang, Jiaoli Liu, Xiaofeng Wang, Dong Zhang, Feng Ke, Haiquan Hu, Wenjun Wang, Bingyuan Zhang, Hongdong Li, Bo Liu, Qinglin Wang

**Affiliations:** ^1^Shandong Key Laboratory of Optical Communication Science and Technology, School of Physics Science and Information Technology, Liaocheng University, Liaocheng, China; ^2^Department of Geological Sciences, Stanford University, Stanford, CA, United States; ^3^State Key Laboratory of Superhard Materials, Jilin University, Changchun, China; ^4^Lab of Functional Molecules and Materials, School of Physics and Optoelectronic Engineering, Shandong University of Technology, Zibo, China

**Keywords:** n-ZnO nanorods, p-degenerated diamond, heterojunction, negative differential resistance, high temperature

## Abstract

In the present study, an n-ZnO nanorods (NRs)/p-degenerated diamond tunneling diode was investigated with regards to its temperature-dependent negative differential resistance (NDR) properties and carrier tunneling injection behaviors. The fabricated heterojunction demonstrated NDR phenomena at 20 and 80°C. However, these effects disappeared followed by the occurrence of rectification characteristics at 120°C. At higher temperatures, the forward current was increased, and the turn-on voltage and peak-to-valley current ratio (PVCR) were reduced. In addition, the underlying mechanisms of carrier tunneling conduction at different temperature and bias voltages were analyzed through schematic energy band diagrams and semiconductor theoretical models. High-temperature NDR properties of the n-ZnO NRs/p-degenerated diamond heterojunction can extend the applications of resistive switching and resonant tunneling diodes, especially in high-temperature, and high-power environments.

## Introduction

Negative differential resistance (NDR) is a non-linear carrier transport phenomenon, whereby the electrical current decreases with increasing bias voltage. Due to its remarkable carrier transport features, NDR has a large potential for device applications, such as resistive switching, logic devices, and oscillators (Malik et al., 2018). NDR involves the quantum transition process related to the p-n junction doping concentration, which mostly occurs in heavily doped degenerated p-n junction structures.

To date, several materials, such as biological molecules (Peng et al., 2009), organic crystal (Yang et al., 2009; Tonouchi et al., 2017), metal-oxide heterojunctions (Ito et al., 2007), and semiconductor quantum wells (Shin and Kim, 2015) have been exploited for NDR devices. Among the materials, zinc oxide (ZnO) has wide application prospects in the field of optoelectronics owing to its wide bandgap, high exciton binding energy, and unintentionally doped n-type semiconductor (Wang et al., 2014b; Sang et al., 2016; Chang et al., 2018). Boron-doped diamond is also widely applied in high-power optoelectronic devices due to its higher hole mobility of p-type semiconductor, larger thermal conductivity, and greater chemical stability (Wang et al., 2017). P-diamond exerts degenerative features through heavy boron doping and displays an NDR phenomenon when combined with n-ZnO (Nishimura et al., 1991; Lee and Pickett, 2006). The NDR phenomenon has been observed for the n-ZnO nanostructures/p-degenerated diamond heterojunction at room temperature, which may be attributed to the tunneling current in its structure (Li et al., 2013). However, it remains an urgent need to assess the possibility of further practical utilization of a tunneling diode device with remarkable characteristics in high-temperature and high-power environments. Therefore, the NDR properties of n-ZnO NRs/p-degenerated diamond-based devices at high temperature still merit further study, and some other questions should be thoroughly explored. For example, how does the temperature affect the interfacial barrier height of the heterojunction as well as the transition, recombination, and tunneling process of the carrier? At high temperatures, how about the variation of NDR properties upon the changes in Fermi level and impurity deep levels?

In the present study, we aimed to investigate the temperature-dependent NDR performance of n-ZnO NRs/p-degenerated diamond tunneling. The variation trends of different heterojunction physical parameters, such as turn-on voltage, forward current, and peak-to-valley current ratio (PVCR), are also examined. Furthermore, the mechanisms of temperature-related charge tunneling transfer and carrier-injected behavior are discussed through the proposed schematic energy band diagram and semiconductor principle.

## Result and Discussion

**Figure 1:**
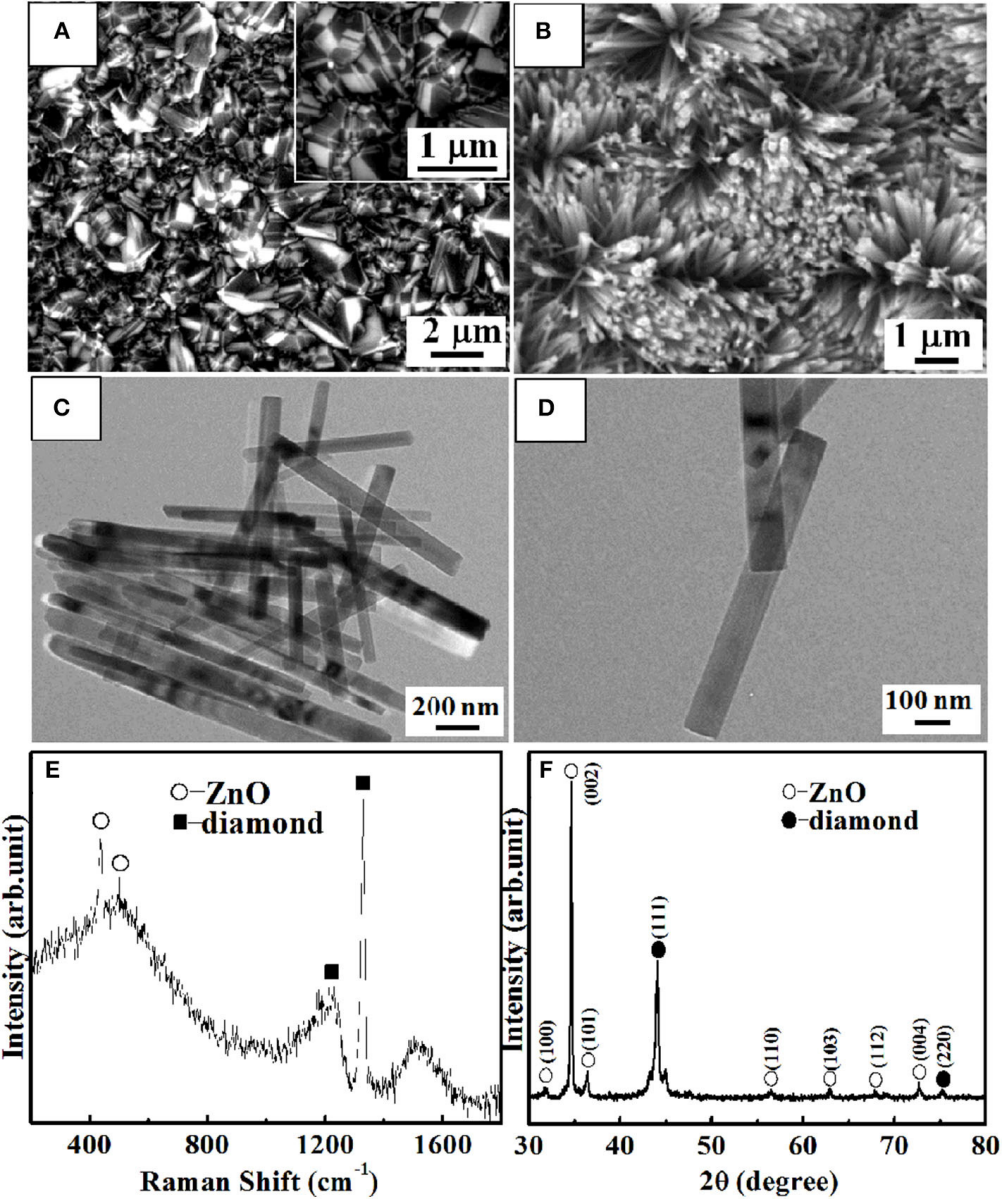
SEM images of p-degenerated diamond **(A)** and n-ZnO NRs **(B)** grown on p-degenerated diamond. TEM images of the as-synthesized n-ZnO NRs at 200-nm **(C)** and 100-nm **(D)** magnifications. Raman spectra **(E)** and XRD patterns **(F)** of n-ZnO NRs/p-degenerated diamond heterojunction.

The representative SEM image of p-degenerated diamond film is displayed in Figure 1A. The average grain size is ~500 nm dominant pyramid-shaped morphology and twinned crystal (inset of Figure 1A) owing to its high level of boron doping. As shown in Figure 1B, ZnO NRs are growing nearly vertically on p-degenerated diamond substrate. It is clearly observed from the TEM (Figures 1C,D) that the ZnO NRs display a large aspect ratio with a smooth surface with the average length and diameter of ~2 μm and ~80 nm, respectively.

Figures 1E,F reveals the Raman spectra and XRD patterns of ZnO NRs grown on a p-degenerated diamond film, respectively. For Raman spectra (Figure 1E), the peaks at 332, 437, and 582 cm^−1^ correspond to the E_2H_-E_1H_, E_1H_, and A_1_(LO) modes of ZnO NRs (Rajalakshmi et al., 2000). It was noted that, due to the introduction of heavily boron-doped diamond (holes carrier concentration was ~1 × 10^20^/cm^3^ as tested by the Hall effect), the p-degenerated diamond peak (1,328 cm^−1^) showed the asymmetry characteristics and downshift to a lower wave number of the zone-centered phonon band. On the other hand, the appearance of the two wide bands indexed at 500 and 1,200 cm^−1^ was attributed to the high level of boron-doped diamond (Li et al., 2010). For XRD patterns (Figure 1F), most peaks were indexed from ZnO hexagonal single crystal mostly along [0001] orientation (Lam et al., 2017) except for the diamond (111) and (220) peaks at 44° and 75.3°. It is noticed that the tiny peaks at 43.3° and 45° correspond to the (010) and (101) planes of hexagonal graphite, respectively, which indicates the existence of a small amount of graphite phase associated with the graphitization of the amorphous carbon, and it is usually detected surrounding the diamond grains.

**Figure 2:**
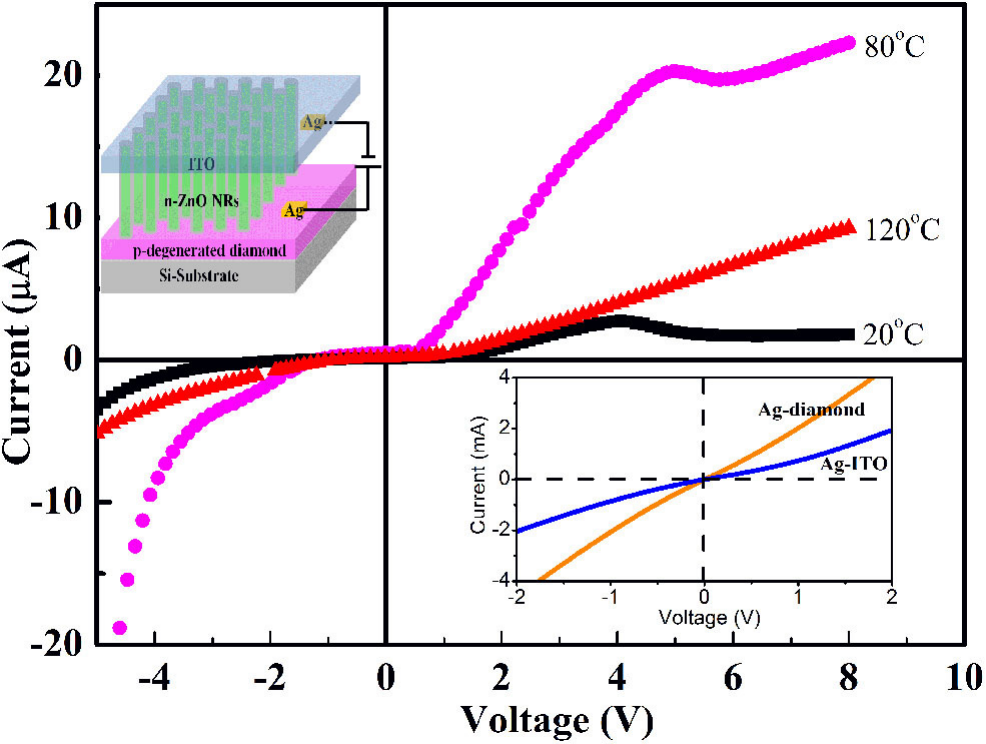
I–V plots of the n-ZnO NRs/p-degenerated diamond at different temperatures of 20~120°C. The top inset shows the schematic diagram of the n-ZnO NRs/p-degenerated diamond heterojunction device. The bottom inset is the linear of I–V characteristics of ohmic contacts of Ag/ITO and Ag/p-degenerated diamond.

The schematic diagram of the n-ZnO NRs/p-degenerated diamond tunneling diode is depicted in the inset of Figure 2. The I–V performances of Ag/ITO and Ag/p-degenerated diamond demonstrate a linear relationship (Figure 2) with an ohmic contact. Similarly, ZnO-ITO is of ohmic contact due to the closely related function (Yang et al., 2008). The resistivity and mobility of the p-degenerated diamond are 10 Ω cm^2^ and 11 cm^2^ V^−1^ s^−1^, respectively, as measured by the Hall effect. The carrier density exhibits a higher concentration of 1.7 × 10^20^ cm^−3^, which is deemed to the degenerated semiconductor.

The I–V characteristics of the n-ZnO NRs/p-degenerated diamond tunneling diode at 20, 80, and 120°C are presented in Figure 2. The results demonstrate an obvious tunneling diode behavior with NDR phenomena at 20 and 80°C. The values of turn-on voltage, peak current, valley current, and PVCR under different temperatures are listed in Table 1. In the forward direction, the I–V plots of the tunneling diode could be divided into three regions: (i) band-to-band tunneling, (ii) excess, (iii) diffusion currents. The current was first increased to the maximum value of peak current I_p_ (from 2.6 μA at 20°C to 20.4 μA at 80°C) with a peak voltage V_p_ (4 V for 20°C and 5 V for 80°C). After that, the current was decreased to a minimum value of valley current I_V_ (from 1.5 μA at 20°C to 19.3 μA at 80°C) with a valley voltage V_V_ (5.1 V at 20°C and 5.8 V at 80°C). The NDR effects with PVCRs of 1.7 at 20°C and 1.1 at 80°C obviously emerged. For the voltages higher than V_v_, the current increased slightly with the forward bias. Meanwhile, the forward turn-on voltages decreased from 1.5 V at 20°C to 0.7 V at 80°C. The turn-on voltage is defined as the voltage at a current density of 0.2 μA (Wang et al., 2017). However, at a higher temperature (120°C), the effect of NDR exhibited a gradually decreasing trend. It was noted that the forward current increased significantly: 10 and 5 times higher at 80 and 120°C, respectively. The reverse-biased region was found with high breakdown current density and showed an increasing trend for large inverse voltages at higher temperatures. The decreased turn-on voltage, increased forward current, and appearance of the NDR effects suggest the relatively high performance of the n-ZnO NRs/p-degenerated diamond tunneling diode at 80 and 120°C.

**Table 1:** Electrical parameters of n-ZnO NRs/p-degenerated diamond heterojunction at 20 and 80°C.

**Temperature (°C)**	**20**	**80**
Peak current (μA)	2.6	20.4
Valley current (μA)	1.5	19.3
Peak-to-valley current ratio	1.7	1.1
Turn on voltage (V)	1.5	0.7

**Figure 3:**
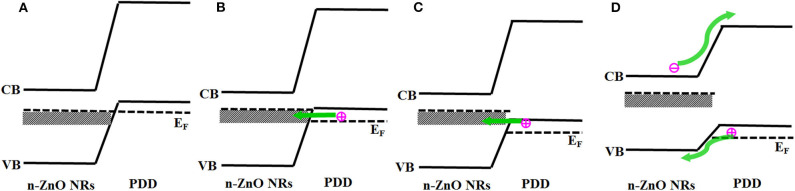
**(A)** Thermal equilibrium at zero bias. **(B)** Forward bias such that peak current is obtained. **(C)** Forward bias approaching valley current. **(D)** Forward bias with diffusion current and no tunneling current.

To explore the mechanism of NDR, a schematic energy band diagram was established at 20°C (Figure 3). Notably, the prepared p-degenerated diamond exhibited a high carrier concentration of 1 × 10^20^/cm^3^, which could be considered as a degenerated structure. For the degenerated p-type diamond, it is speculated that the Fermi level enters into the valence band, and the unoccupied energy states exist near the top of valence band (Lee and Pickett, 2006; Li et al., 2013). As illustrated in Figure 3A, the unintentionally doped ZnO NRs are not degenerated, the Fermi level existed in the band gap of ZnO along with deep level defects forming a broad band, which originated from zinc interstitials and oxygen vacancies (Wang et al., 2014a; Zhao et al., 2016). When the bias voltage is introduced (Figure 3B), the ZnO conduction band and deep levels move up, and the carrier may tunnel from the empty energy states in the valence band of p-degenerated diamond into the deep level band of ZnO, indicating that band-to-band tunneling current and peak current are obtained. If the forward bias is further added (Figure 3C), the bands could be uncrossed, and a lesser number of states are available on the diamond side to be tunneled, leading to a decrease in tunneling current. Consequently, the NDR occurred and is approaching the valley current. With a continuous bias voltage (Figure 3D), the bands are uncrossed, and the regular diffusion current and excess current begin to dominate the diode current.

**Figure 4:**
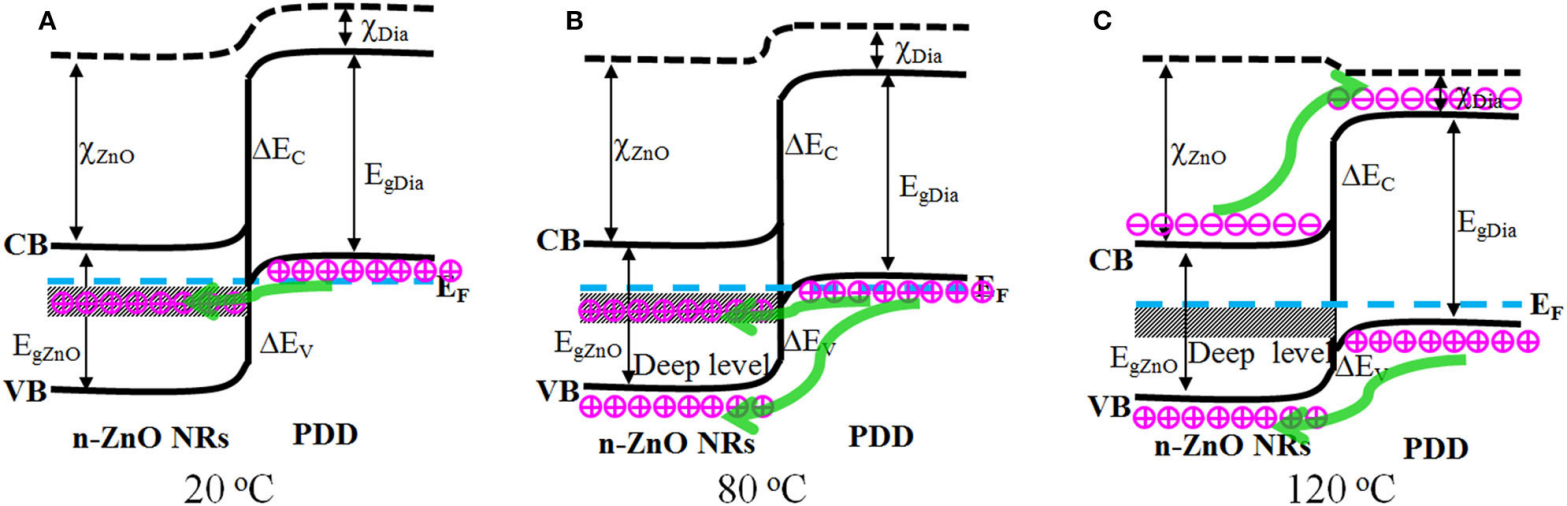
Energy band diagram of n-ZnO NRs/p-degenerated diamond heterojunction at **(A)** 20, **(B)** 80, and **(C)** 120°C.

To elucidate the mechanism underlying the increased peak and valley current and the reduced turn-on voltage in a temperature-dependent manner for 80°C, a more detailed schematic energy band diagram is established at 20, 80, and 120°C (Figure 4). According to Anderson's model (Figure 4A), after the combination of n-ZnO and p-degenerated diamond, the conduction band barrier ΔE_C_ is greater than the valence band barrier ΔE_v_, and the injected current is largely dominated by the hole carriers in the valence band of p-degenerated diamond (Sang et al., 2015). Since the tunneling and excess current are both exponentially dependent on the barrier thickness of the carrier, the increased current at a high temperature of 80°C might be due to the variation of the carrier barrier parameters. At 80°C (Figure 4B), the Fermi level E_F_ of the heterojunction often moves close to the middle position of the bandgap (Kwok and Sze, 2007), both n-ZnO and p-degenerated diamond are approximately proportional to the intrinsic semiconductor, and more intrinsic carriers are excited. The carrier barrier thicknesses of ΔE_C_ and ΔE_V_ in the depletion layer are decreased, and more carrier injection from the ZnO conduction band (diamond valence band) to the diamond conduction band (ZnO valence band), thus, becomes simpler under the forward basis. As a result, the forward peak and valley current in the forward bias scale as well as the turn-on voltage are lower at 80°C for the n-ZnO NRs/p-degenerated diamond heterojunction.

According to the equation of mass-action theory (Kwok and Sze, 2007), the carrier concentration (n_c_) is calculated as follows:

(1) $$\begin{array}{l} n_{c}\approx \sqrt{\frac{N_{D}N_{C}}{2}}\exp\left[-\frac{\left(E_{c}-E_{D}\right)}{2k_{B}T}\right]. \end{array}$$

N_C_ is the effective density of states in the conduction band, *E*_*C*_ − *E*_*D*_ represents the ionization energy for donor, and n_c_ is plotted as a function of the reciprocal temperature. At relatively high temperatures, n_c_ is increased, more electrons and holes are thermally ionized, and a higher defect density originated from oxygen vacancies may exist at the defect-level of ZnO. The forward current can indeed dominate more carrier-resonant tunneling through the oxygen defect band of ZnO to the empty states of the diamond valence band. Therefore, another reason underlying the increased forward current and reduced turn-on voltage at 80°C may be due to a greater variability in oxygen defect densities and a higher number of carrier tunneling processes.

Noticeably, the NDR phenomenon became slightly weak and the PVCR decreased at a higher temperature of 80°C or even disappeared completely at 120°C. Typically, the effect of NDR is observed at lower temperatures, and the carriers can tunnel from the diamond valence band to the ZnO defect band at 20 and 80°C (Figures 4A,B). While at 120°C, PVCR is reduced and, thus, leads to the disappearance of NDR due to enhanced thermionic emission current and variation of energy band (Kathalingam et al., 2015). As shown in the schematic energy band diagram (Figure 4C), the Fermi level moved up and entered into the bandgap at 120°C. The n-ZnO NRs and p-degenerated diamond semiconductor were approaching the intrinsic semiconductor. Consequently, there was no filled empty energy state that existed for either ZnO or diamond at 120°C. Moreover, the tunneling current was no longer injected from the p-diamond valence band to the ZnO deep level band. The regular diffusion current and excess current injected from the diamond valence band (ZnO conduction band) to the ZnO valence band (diamond conduction band) conferred a major effect. As a result, the diode switched to a common non-degenerate p-n heterojunction without NDR phenomenon and PVCR at 120°C.

**Figure 5:**
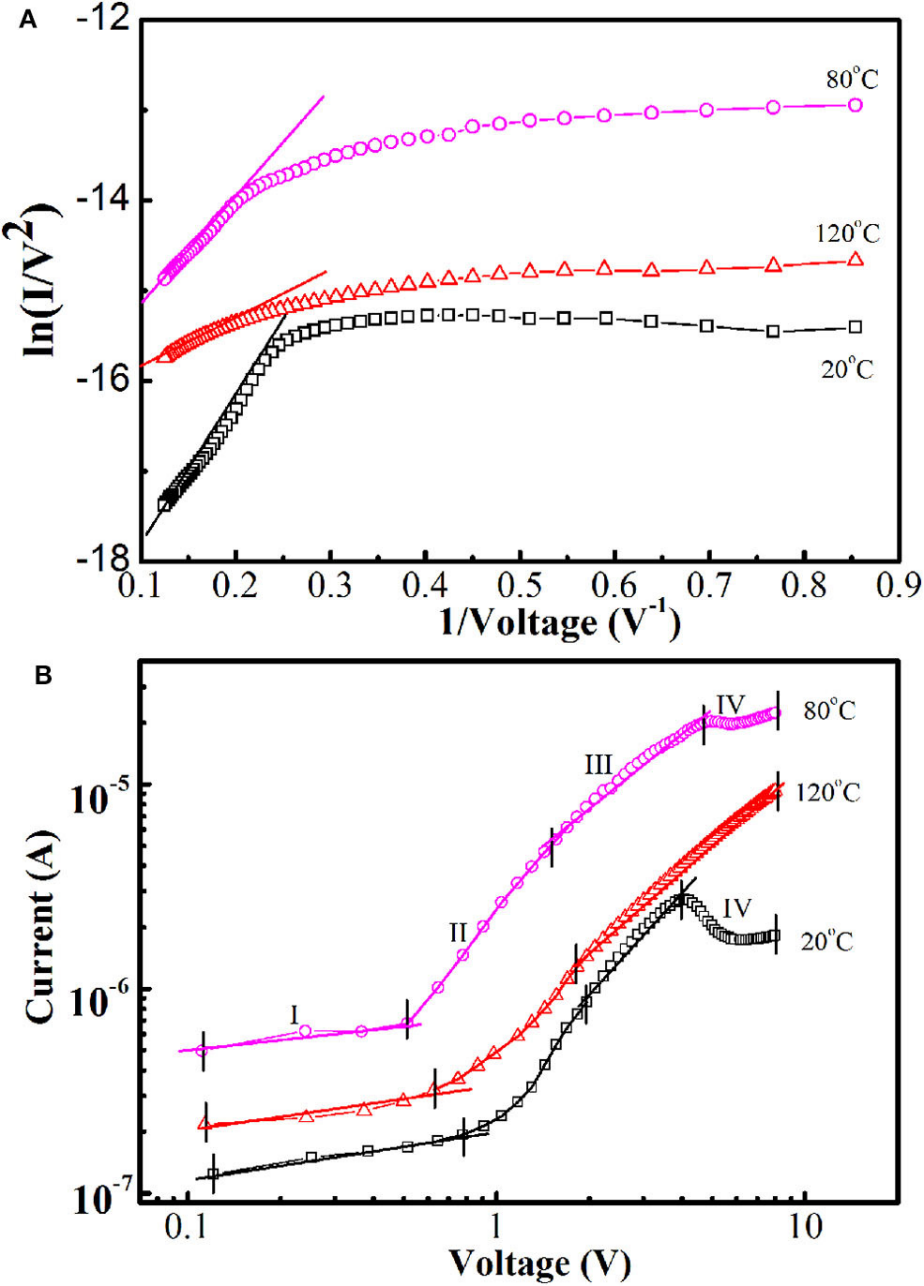
**(A)** Plots of ln (I/V^2^ vs. 1/V). **(B)** log I–log V plots of n-ZnO NRs/p-degenerated diamond heterojunction at 20–120°C.

For a deeper understanding of the tunneling conduction process, I–V properties at different temperatures were investigated based on the Fowler–Nordheim (FN) theory. In a low bias region, the properties adhered to the direct tunneling; when the voltage achieved a higher bias, the conduction model could change to FN tunneling (Malik et al., 2018). According to the FN equation, the current was calculated as follows:

(2) $$\begin{array}{l} I\propto V^{2}\exp\left[\frac{-4d{\left(q\phi \right)}^{{\;}^{3}{/}_{2}}{\left(2m^{*}\right)}^{{\;}^{1}{/}_{2}}}{3q\hbar V}\right], \end{array}$$

where φ is the energy barrier height, d indicates the tunneling distance, and m^*^ is the reduced mass of the charge carrier. The plot of ln(*I*/*V*^2^) vs. 1/V (Figure 5A) in the high-voltage range (4–8 V) displays a linear relationship, suggesting that the conduction mechanism is dominated by FN tunneling. In addition, the non-linear electrical transport phenomena in low bias adhered to the direct tunneling conduction.

Figure 5B shows the log I–log V plots of the device at different temperatures, reflecting the electrical transport behavior. The plot of 120°C is divided into three different regions, and the plots of 20 and 80°C are divided into four regions. In the extremely low bias region I, the transportation of current obeys I~V^0.28^, I~V^0.32^, and I~V^0.36^ for 20, 80, and 120°C, respectively. The exponent values increased, and more approached one at higher temperatures, indicating the transport mechanisms may follow ohmic law. In region II, the injected current follows the relationship of I~exp (αV); the fitted values of the injection efficiency α are 1.34 (20°C), 1.71 (80°C), and 1.16 (120°C), which are close to the value (1.5) of ideal vacuum diode acquired in the wide bandgap p-n heterojunctions to the recombination tunneling principle. The higher injection efficiency α at 80°C indicates the more thermally excited carriers appeared and injected at middle voltages. In high bias region III, the I–V plots followed power law of I~V^1.87^, I~V^1.06^, and I~V^1.23^ for 20, 80, and 120°C, respectively. The exponent value at 20°C was close to two, indicating the I–V relationship approaches the space-charge-limited current (SCLC) electric model at this temperature. At 80 and 120°C, the current transmission approach follows a linear relationship of ohmic behavior with the exponential value of approximately one (Hu et al., 2011). Because the edge of the n-ZnO conduction band is exactly opposite to the top of the p-diamond valence band at high voltage scale of 80 and 120°C (Figures 4B,C), there are no available states opposite the filled states. Consequently, the thermal emission carriers might no longer flow and tunnel from the oxygen defect level band of n-ZnO to the unoccupied states of p-degenerated diamond in region III. In region IV, at 20 and 80°C, the I–V plots followed the I-(-V) relationship, indicating the expansion of FN tunneling impeded the SCLC mechanism.

## Conclusions

In summary, an n-ZnO NRs/p-degenerated diamond NDR tunnel device was successfully fabricated. The tunneling current from diamond valence band to deep levels lowered the turn-on voltage and promoted the NDR phenomenon at high temperatures. The forward current at 80 and 120°C were more than 10 and 5 times higher than 20°C, respectively. The temperature-dependent charge transfer mechanism was explored through schematic energy band diagrams and semiconductor features. It was proposed that the n-ZnO NRs/p-degenerated diamond NDR tunnel device exhibited good performance under high-temperature conditions. The findings of this study could provide essential insight into the relevant NDR mechanism and establish general guidelines for designing or optimizing new-type NDR devices at high temperature and other harsh environments.

## Experimental Method

P-degenerated diamond film was synthesized by the 150 V bias-assisted hot filament chemical vapor deposition (HFCVD) method. The silicon wafers (1 cm×1 cm) were abraded by diamond paste for nucleation enhancement and cleaned ultrasonically using ethanol solution. A spiral tantalum wire was used as a filament, and its heating temperature was about 2,000°C. The main growth parameters were as follows: total pressure = 40 Torr, CH_4_/H_2_ flow rate = 2.6/200 sccm, and substrate temperature = ~700–800°C. Liquid B(OCH_3_)_3_ incorporated to the boron source carried bubbling H_2_ gas with a flow rate of 20 sccm. The diamond films displayed a thickness of 4 μm with a deposition time of 4 h. The ZnO NRs fabricated on p-degenerated diamond film were synthesized in a quartz tube within a horizontal tube furnace via the thermal evaporation method. The mixed raw powders of ZnO and aluminum were heated in the quartz tube at 850°C. P-degenerated diamond substrates were placed downstream from the source to the other end of the tube at ~500°C. The experiments were performed under a constant pressure of 6 × 10^4^ Pa. After evaporation and growth, the samples were drawn out and cooled to room temperature (Sang et al., 2012, 2015, 2018). To obtain the n-ZnO NRs/p-degenerated diamond tunneling diode, a transparent conductive indium-tin-oxide (ITO) glass was pressed on the top of ZnO NRs as the negative electrode and a silver (Ag) wire was employed as the positive electrode for p-degenerated diamond.

The morphologies and structures of the obtained p-degenerated diamond and ZnO were characterized using a scanning electron microscope (SEM, JEOL JXA 8200). Transmission electron microscope (TEM) images were taken by JEM-2100 transmission electron microscope at an operating voltage of 200 KV. Raman microscopy (by a Renishaw in Via 514.5 nm line of an Ar^+^ ion laser) and X-ray diffraction (XRD, Rigaku D/max-RA with Cu Kα radiation of l =1.54056 Å) were also carried out. The temperature-dependent NDR characteristics of the n-ZnO NRs/p-degenerated diamond tunneling diode were determined by Keithley Series 2400 SourceMeter Instruments.

## Data Availability Statement

All datasets generated for this study are included in the article/supplementary material.

## Author Contributions

QW and DS conceived the idea and measured the electrical transport properties and analyzed the result data. DS, JL, XW, and DZ fabricated the samples and devices and measured XRD, SEM, TEM, and Raman spectra. DS, QW, and FK wrote the manuscript. HH, WW, BZ, HL, and BL help to analyze the data. All authors have read and approved the final manuscript.

## Conflict of Interest

The authors declare that the research was conducted in the absence of any commercial or financial relationships that could be construed as a potential conflict of interest.
